# Prognostic and predictive value of *TP53 *mutations in node-positive breast cancer patients treated with anthracycline- or anthracycline/taxane-based adjuvant therapy: results from the BIG 02-98 phase III trial

**DOI:** 10.1186/bcr3179

**Published:** 2012-05-02

**Authors:** Lynnette Fernández-Cuesta, Catherine Oakman, Priscila Falagan-Lotsch, Ke-seay Smoth, Emmanuel Quinaux, Marc Buyse, M Stella Dolci, Evandro De Azambuja, Pierre Hainaut, Patrizia Dell'Orto, Denis Larsimont, Prudence A Francis, John Crown, Martine Piccart-Gebhart, Giuseppe Viale, Angelo Di Leo, Magali Olivier

**Affiliations:** 1 Molecular Carcinogenesis Group, International Agency for Research on Cancer, 150 Cours Albert Thomas, 69372 Lyon Cedex 8, France; 2 'Sandro Pitigliani' Medical Oncology Unit, Hospital of Prato, Istituto Toscano Tumori, Piazza dell'Ospedale 2, 59100 Prato, Italy; 3 International Drug Development Institute, Avenue Provinciale 30, 1340 Louvain-La-Neuve, Belgium; 4 Breast European Adjuvant Studies Team, Institut Jules Bordet, Université Libre de Bruxelles, Boulevard de Waterloo 121, 1000 Brussels, Belgium; 5 University of Milan School of Medicine and European Institute of Oncology, Via Ripamonti 435, 20141 Milan, Italy; 6 Institut Jules Bordet, Université Libre de Bruxelles, Boulevard de Waterloo 121, 1000 Brussels, Belgium; 7 Peter MacCallum Cancer Centre, St. Andrews Place, East Melbourne, Victoria 3002, Australia; Australia and New Zealand Breast Cancer Trials Group, University of Newcastle, Newcastle, NSW 2310, Australia; International Breast Cancer Study Group, Effingerstrasse 40, 3008 Bern, Switzerland; 8 Irish Clinical Oncology Research Group, 60 Fitzwilliam Square, Dublin, 2, Ireland; 9 Department of Medical Oncology, Institut Jules Bordet, Université Libre de Bruxelles, 121 Boulevard de Waterloo, 1000 Brussels, Belgium

## Abstract

**Abstract:**

**Introduction**

Pre-clinical data suggest p53-dependent anthracycline-induced apoptosis and p53-independent taxane activity. However, dedicated clinical research has not defined a predictive role for *TP53 *gene mutations. The aim of the current study was to retrospectively explore the prognosis and predictive values of *TP53 *somatic mutations in the BIG 02-98 randomized phase III trial in which women with node-positive breast cancer were treated with adjuvant doxorubicin-based chemotherapy with or without docetaxel.

**Methods**

The prognostic and predictive values of *TP53 *were analyzed in tumor samples by gene sequencing within exons 5 to 8. Patients were classified according to p53 protein status predicted from *TP53 *gene sequence, as wild-type (no *TP53 *variation or *TP53 *variations which are predicted not to modify p53 protein sequence) or mutant (p53 nonsynonymous mutations). Mutations were subcategorized according to missense or truncating mutations. Survival analyses were performed using the Kaplan-Meier method and log-rank test. Cox-regression analysis was used to identify independent predictors of outcome.

**Results**

*TP53 *gene status was determined for 18% (520 of 2887) of the women enrolled in BIG 02-98. *TP53 *gene variations were found in 17% (90 of 520). Nonsynonymous p53 mutations, found in 16.3% (85 of 520), were associated with older age, ductal morphology, higher grade and hormone-receptor negativity. Of the nonsynonymous mutations, 12.3% (64 of 520) were missense and 3.6% were truncating (19 of 520). Only truncating mutations showed significant independent prognostic value, with an increased recurrence risk compared to patients with non-modified p53 protein (hazard ratio = 3.21, 95% confidence interval = 1.740 to 5.935, *P *= 0.0002). p53 status had no significant predictive value for response to docetaxel.

**Conclusions**

p53 truncating mutations were uncommon but associated with poor prognosis. No significant predictive role for p53 status was detected.

**Trial registration:**

ClinicalTrials.gov NCT00174655

## Introduction

One of the commonest genetic lesions in breast cancer is mutation of the tumor suppressor gene *TP53*, encoding the p53 protein. p53 is a transcription factor that mediates antiproliferative mechanisms in response to various forms of cellular stresses, in particular DNA damage [1]. Different types of DNA damage activate p53 through different pathways, resulting in different responses including senescence, cell-cycle arrest and apoptosis [2].

Experimental models of breast cancer also show that mutation of p53 may confer an aggressive tumor behavior that is not seen in p53-null models [3]. Most mutant p53 proteins lose their ability to bind wild-type p53 responsive elements and to regulate the expression of p53 transcriptional targets, thus losing tumor suppressor activity. However, cellular preservation of mutated p53 may confer malignant potential such as the capacity to metastasize, through gains of function activities (reviewed in [4] Oren and Rotter, 2010).

*TP53 *mutation is generally associated with a poor prognosis, predicting poor disease-free survival (DFS) and overall survival (OS) in breast cancer patients [5,6]. As a predictive biomarker for treatment response, the role of p53 remains a matter of debate. More than a decade ago, p53 emerged as an important factor in the activity of DNA-damaging chemotherapies [7]. Indeed, preclinical studies suggested p53-dependent anthracycline-induced apoptosis and p53-independent taxane activity [7,8]. Many clinical studies undertaken in the last decade have sought to validate these results. Most studies have retrospectively assessed p53 in subgroups from biologically unselected breast cancer trials [9-13]. Clinical data remains conflicting and inconclusive, and no robust predictive correlation has surfaced. An important recent study is the neoadjuvant phase III EORTC 10994/BIG 00-01 trial, which is the only study to be prospectively powered to assess the predictive role of p53 [14]. p53 status was assessed using an RNA-based technique, which detects functionally important p53 mutations using a yeast-based assay [15]. The prognostic role of p53 was confirmed, but p53 was not predictive of response or resistance to docetaxel.

The methods used to evaluate *TP53 *status and the diversity of observed mutations constitute sources of heterogeneity when analyzing the clinical impact of mutations. More than 75% of *TP53 *mutations are missense mutations that produce mutant proteins, and up to 25% of mutations are small insertions or deletions that produce truncated proteins. Determination of p53 status by immunohistochemistry (IHC) is plagued by high false-positive rates (overexpression of p53 wild-type protein), high false-negative rates (truncating mutations that stain negative), and a poor level of correlation with *TP53 *gene mutations [9]. IHC has been surpassed by direct DNA sequencing, functional assays in yeast and p53 genetic signatures. Studies that have used gene resequencing to assess *TP53 *status have produced more consistent results for the prognostic value of mutations [5,16]. However, results of gene resequencing should be interpreted in terms of downstream p53 protein functions as *TP53 *gene mutations impact differently on protein functions, as evidenced in functional assays in yeast or human cells [17,18]. Indeed, assessment of the transactivation capacity of p53 mutant proteins on different p53 target sequences has shown great variability of activities between mutant proteins and target sequences. Whereas hotspot missense mutations in the DNA-binding domain lead to a general loss of specific transactivation capacity on all target sequences, missense mutations outside the DNA-binding domain more often retain transcriptional activity on some promoters. Moreover, some mutant proteins may have dominant-negative effects on wild-type p53 or exert pro-oncogenic effects independently of wild-type p53 [19]. The *TP53 *Function Database created at the International Agency for Research on Cancer (IARC), Lyon, France, allows the classification of *TP53 *mutations according to their predicted impact on p53 protein activities [19].

In the late 1990s, the Breast International Group (BIG) 02-98 phase III randomized trial was one of many clinical trials launched to explore the role of adjuvant taxanes in early breast cancer (http://ClinicalTrials.gov Identifier: NCT00174655) [20]. Improved outcomes from the addition of taxanes to anthracycline-based therapy have been reported in many [20-25] but not all trials [26-28]. A recent meta-analysis reported superior outcomes from the addition of a taxane to anthracycline-based regimens in high-risk disease [29]. The absolute benefit deriving from the addition of taxanes is modest, less than 10% absolute improvement in DFS. A key issue for clinical practice is that despite taxane benefit for clinical trial cohorts, there is no predictive marker for the identification of individuals that are most likely to benefit from taxane.

With this background of inconsistent data for clinical utility of p53 status and absence of predictive markers for taxane use, we undertook a retrospective, exploratory analysis in BIG 02-98 to investigate the prevalence, and prognostic and predictive value of different types of *TP53 *mutations. Mutation classifications based on the IARC *TP53 *Function Database were used to estimate the functional impact of *TP53 *gene variations, rather than their presence per se.

## Materials and methods

### Study population

The current study is a retrospective study on primary tumor samples from a larger population of patients participating in the clinical trial BIG 02-98. Details of patients and methods in this trial have been described previously by Francis *et al. *[20]. Briefly, BIG 02-98 was a multicenter, randomized phase III adjuvant trial of 2887 women aged 18 to 70 years with operable, clinical stage T1 to T3 invasive breast adenocarcinoma, with at least one positive axillary lymph node. Between 1998 and 2001, patients were randomly assigned at a ratio of 1:1:2:2 to one of the following four adjuvant chemotherapy regimen arms*: Arm A*: sequential control = four cycles of doxorubicin (A) 75 mg/m^2^, followed by three cycles of classical cyclophosphamide, methotrexate and 5-fluorouracil (CMF); *Arm AC*: concurrent control = four cycles of AC 60/600 mg/m^2^, followed by three cycles of CMF; *Arm A-T*: sequential docetaxel = three cycles of A 75 mg/m^2 ^followed by three cycles of docetaxel (T) 100 mg/m^2 ^followed by three cycles of CMF; *Arm AT*: concurrent docetaxel = four cycles of AT 50/75 mg/m^2 ^followed by three cycles of CMF. The planned cumulative doxorubicin dose was higher in the control arms (A: 300 mg/m^2^; AC: 240 mg/m^2^) than in the docetaxel arms (A-T: 225 mg/m^2^; AT: 200 mg/m^2^). A-T and AT had different docetaxel dose intensity but the same cumulative dose (300 mg/m^2^). Endocrine therapy and radiotherapy were administered according to local guidelines. Adjuvant trastuzumab was not available. Institutional Ethics Committees approved the protocol at all participating sites and patients provided written informed consent.

### p53 substudy cohort

From BIG 02-98, patients who had formalin-fixed paraffin-embedded (FFPE) primary tissue submitted centrally and for which there was sufficient remaining tumor tissue for *TP53 *gene analysis were selected for the p53 biomarker study. A total of 666 cases were selected, of whom 520 were successfully analyzed for exons 5 to 8. The substudy population was representative of the entire BIG-02-98 population for all baseline patient and tumor characteristics. [See Additional file 1.] The translational study was approved by the Ethics Committee of the Jules Bordet Institute (IJB) in Brussels, Belgium, which served as the coordinating center for this retrospective study.

### Tumor material and immunohistochemistry

The study protocol requested central collection of one FFPE tumor sample for each patient. Slides were prepared by the Pathology Department at IJB and sent to the European Institute of Oncology (EIO), Milan, Italy for central pathology analyses and genomic DNA extraction. Slide review, IHC and fluorescence in situ hybridization (FISH) were performed on whole tissue sections from FFPE samples. Tumor grade was centrally reviewed. Unstained tumor specimens were stained for estrogen receptor (ER), progesterone receptor (PgR), human epidermal growth factor receptor 2 (HER2) and Ki-67 (all specific monoclonal or polyclonal (for HER2) antibodies were purchased from Dako, Glostrup, Denmark). IHC results were reported as the percentage of invasive tumor cells showing definite immunoreactivity. FISH was performed for HER2 according to the manufacturer's instructions (Abbott-Vysis Inc., Downers Grove, IL, USA). Thresholds for positivity were defined as: ER: ≥ 1%; PgR: ≥ 1%, HER2: IHC 3+ (more than 10% invasive tumor cells with intense and circumferential membrane staining) or 2+ and FISH positive (HER2:CEP17 ratio > 2). The Ki-67 threshold used in the distinction of luminal A and luminal B subtypes was defined as ≥ 14%, based on published work by Cheang *et al. *[30].

Four breast cancer subtypes were defined using central laboratory defined parameters, as follows: (1) highly endocrine responsive (luminal A): ER-positive, PgR-positive, HER2-negative and Ki-67 low; (2) incompletely endocrine responsive (luminal B): ER-positive and PgR-negative, independent of other parameters, or ER-positive, PgR-positive and at least one of grade 3, HER2-positive and/or Ki-67 high; (3) HER2-positive: ER-negative, PgR-negative and HER2-positive; and (4) triple-negative: ER-negative, PgR-negative and HER2-negative. [See Additional file 2.]

### DNA extraction

Genomic DNA was extracted from two to three serial sections of FFPE-archived specimens (10 μm thick). Dewaxing was obtained by xylene and ethanol with alternating vortexing and centrifuging. DNA was then extracted with a commercially available kit (DNeasy Blood & Tissue Kit, Qiagen N.V., Venlo, The Netherlands). Tissue was lysed overnight with proteinase K digestion in denaturing condition and DNA bound to spin column silica membrane. The contaminants were washed away and DNA was eluted with 100 μL of sterile distilled water. The final DNA concentration was evaluated by OD260 (GeneQuant II, Pharmacia Biotech, Uppsala, Sweden).

### *TP53 *mutation screening and sample classifications

Genomic DNA was screened for *TP53 *mutations at IARC. Exons 5 to 8 of *TP53 *were analyzed in all available samples by polymerase chain reaction (PCR). Exon 4 was analyzed in a subset of samples with sufficient available tumor material. Direct sequencing of genomic DNA has been described in the IARC protocol [31]. Mutations were screened on both DNA strands and were confirmed in an independent PCR product.

*TP53 *gene sequencing reported all variations. These variations were used to classify samples according to p53 protein status, which take into account the predicted impact of genetic variations on p53 proteins. Thus, a variation is considered a mutation when it is predicted to modify p53 protein sequence. Tumors were classified as wild-type p53 or mutated p53. (See Table 1.)

**Table 1:** p53 mutation status and prevalence.

p53 status	Rules	Prevalence (%) N = 520
Wild-type p53	No variation*; synonymous variations; intronic variations outside splice sites	435 (83.6%)

Mutated p53	Any variation predicted to modify protein sequence: missense, nonsense, insertion, deletion, variation in splice sites	85 (16.3%)

Subcategorization of mutated p53		

Missense mutation	Protein changed by one amino acid	64 (12.3%)

Truncating mutation	Nonsense, insertion**, deletion**, variation in splice sites	19 (3.6%)

Not classified***		2 (0.4%)

Sample classification status is based on the predicted impact of mutation on p53 protein. Only exons 5 to 8 and splicing junctions were sequenced.

*Any nonsynonymous variation in exon 5 to 8. **All insertion and deletion found in this series induced a frameshift.. ***Not subclassified because the sequencing result was missing for one exon.

Mutated p53 protein status was further subcategorized as missense or truncating. (See Table 1.) Missense mutations produce proteins that are changed by one amino acid. Truncating mutations include nonsense, frameshift insertions and deletions, and variations in consensus splice sites. Annotations from the IARC *TP53 *Database [19] were used to also stratify missense mutations according to their transactivation activities in yeast functional assays, or to their position inside or outside DNA-binding motifs (L1/L2/L3 loops) [17,32]. Sample subclassification was done when sequencing result was obtained for all exons 5 to 8. Two samples were not subclassified because the sequencing result was missing for one exon.

### Statistical analyses

The study was based on the hypotheses that p53 mutated tumors would have the worst clinical outcome and the largest benefit from the addition of docetaxel. Statistics were performed using SAS version 9.1. (SAS Institute Inc., Cary, NC, USA) and Minitab version 13 software (Minitab Inc., State College, PA, USA). The chi-square test was used to compare the distributions of clinicopathological parameters by randomized treatment arm (anthracycline control arms, A and AC, versus taxane arms, A-T and AT), and by p53 status (wild-type p53 versus p53 mutated). Fisher's exact test was used to compare the distributions of clinicopathological parameters by p53 mutation subcategorization (wild-type p53 versus missense mutation; wild-type p53 versus truncating mutation).

DFS was calculated from the date of randomization to the date of disease recurrence, second primary cancer or death from any cause. OS was calculated from the date of randomization to the date of last follow-up or death from any cause. Survival curves were estimated using the method of Kaplan-Meier and curves for different classifications were compared using the log-rank test. Multivariate Cox regression models, with backward selection, were used to test the prognostic effect of p53 status after adjusting for other important prognostic variables. Multivariate DFS analysis included patient characteristics (age, menopausal status, body mass index (BMI)), tumor characteristics (tumor size, number of positive lymph nodes, histopathological type, histological grade, IHC defined subtypes, p53 mutation status) and treatment characteristics (mastectomy, radiotherapy, use of tamoxifen, chemotherapy arm (A+AC versus A-T+AT arms), chemotherapy schedule (sequential or concurrent)).

## Results

### Results of BIG 02-98

After an 8-year median follow-up, the second efficacy results of BIG 02-98 did not show significant improvement in DFS from the incorporation of docetaxel compared with the doxorubicin-based control (hazard ratio (HR) = 0.91, 95% confidence interval (CI) = 0.80 to 1.05, *P *= 0.187). However, sequential A-T significantly improved DFS compared with the sequential control arm A (HR = 0.81, 95% CI = 0.67 to 0.99, *P *= 0.036), and significantly improved both DFS (HR = 0.84, 95% CI = 0.72 to 0.99, *P *= 0.035) and OS (HR = 0.79, 95% CI = 0.65 to 0.98, *P *= 0.028) compared with concurrent AT [33].

### p53 substudy cohort

From BIG 02-98, 2172 of 2887 (75%) patients had FFPE primary tissue submitted centrally. Of these 2172 patients, 666 patients had sufficient remaining tumor tissue for *TP53 *gene analysis, of whom 520 (18% of the original trial population) were successfully analyzed for exons 5 to 8. Of the 520 tumors, 116 were also analyzed for exon 4. The remaining 142 samples could not be analyzed due to poor DNA quality.

The substudy population was representative of entire BIG-02-98 population for all baseline patient and tumor characteristics. [See Additional file 1.] DFS was similar for patients included and not included in this substudy. [See Additional file 3.]

The majority of patients in this p53 substudy were less than 50 years old, premenopausal and nonobese. (See Table 2.) Most tumors were infiltrating ductal carcinoma, ≤ 20 mm, grade 2 or 3, ER-positive and HER2-negative. Regarding the IHC-defined breast cancer subtypes, most tumors were classified as luminal B, of which, 59 were positive for HER2 (19%, not shown). Between the control (A + AC) and docetaxel (A-T + AT) arms, characteristics differed for ER/PgR status and IHC-defined subtypes: a significantly higher proportion of ER- and PgR-negative tumors were present in the control arms (*P *= 0.046). In keeping with this, there was a trend for a higher proportion of the HER2 subtype and triple-negative subtype in the control arms (*P *= 0.052).

**Table 2:** Characteristics of patients and samples in the p53 substudy.

		Arm (A+AC) Anthracycline Control Arms N = 167	Arm (AT+A-T) Docetaxel Arms N = 353	Total N = 520	*P *value (Chi-square Test)*
Age (years)	Median	48	49	49	0.193
		
	Range	27-66	26-70	26-70	
		
	< 35	12 (7.2%)	22 (6.2%)	34 (6.5%)	
		
	35-49	88 (52.7%)	167 (47.3%)	255 (49.0%)	
		
	50-65	65 (38.9%)	148 (41.9%)	213 (41.0%)	
		
	> 65	2 (1.2%)	16 (4.5%)	18 (3.5%)	

Menopausal status	Premenopausal	104 (62.3%)	212 (60.0%)	316 (60.7%)	0.628
		
	Postmenopausal	63 (37.7%)	141 (39.9%)	204 (39.3%)	

Body Mass Index (BMI)	Nonobese (BMI < 30)	135 (80.8%)	283 (80.2%)	418 (80.4%)	0.858
		
	Obese (BMI > = 30)	32 (19.2%)	70 (19.8%)	102 (19.6%)	

Histopathology	Infiltrating ductal ca.	139 (83.2)	285 (80.7%)	424 (81.5%)	0.441
		
	Infiltrating lobular ca.	13 (7.8%)	40 (11.3%)	53 (10.2%)	
		
	Other	15 (8.9%)	28 (7.9%)	43 (8.3%)	

Tumor size (pT)	T1	55 (32.9%)	96 (27.2%)	151 (29.0%)	0.397
		
	T2	102 (61.1%)	224 (63.4%)	326 (62.7%)	
		
	T3	9 (5.4%)	29 (8.2%)	38 (7.3%)	
		
	Missing	1 (0.6%)	4 (1.1%)	5 (0.9%)	

Number of positive lymph nodes	1-3	83 (49.7%)	189 (53.5%)	272 (52.3%)	0.610
		
	≥ 4	84 (50.3%)	164 (46.4%)	248 (47.7%)	

Histopathologic grade	G1	9 (5.4%)	27 (7.6%)	36 (6.9%)	0.204
		
	G2	77 (46.1%)	153 (43.3%)	230 (44.2%)	
		
	G3	76 (45.5%)	170 (48.1%)	246 (47.3%)	
		
	Missing	5 (3.0%)	3 (0.8%)	8 (1.5%)	

ER/PgR status	ER+/PgR+	106 (63.5%)	265 (75.1%)	371 (71.3%)	0.046
		
	ER+/PgR-	12 (7.2%)	20 (5.7%)	32 (6.1%)	
		
	ER-/PgR-	40 (23.9%)	58 (16.4%)	98 (18.8%)	
		
	Other/missing	9 (5.4%)	10 (2.8%)	19 (3.6%)	

HER2 status	HER2-	126 (75.4%)	288 (81.6%)	414 (79.6%)	0.113
		
	HER2+	33 (19.7%)	58 (16.4%)	91 (17.5%)	
		
	Missing	8 (4.8%)	7 (1.9%)	15 (2.9%)	

IHC subtypes**	Luminal A	28 (16.8%)	56 (15.9%)	84 (16.1%)	0.052
		
	Luminal B	88 (52.7%)	227 (64.3%)	315 (60.6%)	
		
	HER2	15 (8.9%)	17 (4.8%)	32 (6.2%)	
		
	Triple-negative	25 (14.9%)	41 (11.6%)	66 (12.7%)	
		
	Missing	11 (6.6%)	12 (3.4%)	23 (4.4%)	

p53 status	Wild-type	134 (80.2%)	301 (85.3%)	435 (83.6%)	0.147
		
	Mutated	33 (19.8%)	52 (14.7%)	85 (16.3%)	

Hormonotherapy use	Received tamoxifen	118 (70.6%)	274 (77.6%)	392 (75.4%)	0.085
		
	Did not receive tamoxifen	49 (29.4%)	79 (22.4%)	128 (24.6%)	

*Comparison of anthracycline control arms and taxane arms. **See Additional file 2 for definition of IHC subtypes. A, doxorubicin; C, cyclophosphamide; ER, estrogen receptor; IHC, immunohistochemistry; PgR, progesterone receptor: T, docetaxel.

### p53 mutations and sample characteristics

A total of 96 variations within exon 5 to 8 were found in 90 samples (90 of 520, 17%). [See Additional file 4 for full data.] In the 90 patients with a *TP53 *gene variation, 85 patients had only one variation, four patients had two variations, and one patient had three variations. Most variations were missense and were located at classical hotspot codons, except for one specific mutation at codon 259 (p.D259N) that was found in six samples. It is of note that no in-frame deletions or insertions were found in these series. In the patient with three gene variations, each of the three variations was located in introns and was predicted to not affect protein sequence (not shown).

*TP53 *gene status was used to predict p53 protein status and classify patients as wild-type or mutated. Nonsynonymous mutations were further distinguished as missense or truncating. (See Table 1.)

The presence of a mutated p53 protein was associated with older age, postmenopausal status, ductal morphology, higher tumor grades and ER/PgR negativity. (See Table 3.) A strong correlation between IHC-defined subtypes and p53 status was found: the proportion of p53 mutated samples in each IHC subtype showed that the HER2 and triple-negative subtypes had the highest rates of p53 mutations, with 22% (7 of 32) and 36% (24 of 66) of mutated samples respectively, compared to 10% (8 of 84) in the luminal A subtype and 13% (45 of 315) in the luminal B subtype.

**Table 3:** Associations between p53 protein status and clinicopathological parameters.

		Wild-type p53 N = 435	p53 mutated* N = 85	*P *value**	Missense mutation N N = 64****	*P *value***	Truncating mutation N = 19****	*P *value***
Age	< 35	31 (7.1%)	3 (3.5%)	0.0045	2 (3.1%)	0.0061	1 (5.2%)	0.6432
						
	35-49	219 (50.3%)	36 (42.3%)		26 (40.6%)		10 (52.6%)	
						
	50-65	175 (40.2%)	38 (44.7%)		29 (45.3%)		7 (36.8%)	
						
	> 65	10 (2.3%)	8 (9.4%)		7 (10.9%)		1 (5.2%)	

Menopausal status	Premenopausal	248 (57.0%)	38 (44.7%)	0.0768	27 (42.2%)	0.0800	11 (57.9%)	0.7279
						
	Postmenopausal	163 (37.5%)	43 (55.3%)		33 (51.6%)		8(42.1%)	
						
	Not known	24 (5.5%)	4 (4.7%)		4 (6.3%)		0	

Body Mass Index (BMI)	Nonobese (< 30)	352 (80.9%)	66 (77.6%)	0.487	52 (81.3%)	1.0000	12 (63.2%)	0.0744
						
	Obese (> = 30)	83 (19.1%)	19 (22.3%)		12 (18.8%)		7 (36.8%)	

Histopathology	Infiltrating ductal ca.	347 (79.8%)	77 (90.6%)	0.0256	57 (89.1%)	0.0866	18 (94.7%)	0.2922
						
	Infiltrating lobular ca.	51 (11.7%)	2 (2.3%)		2 (3.1%)		0	
						
	Other	37 (8.5%)	6 (7.1%)		5 (7.8%)		1 (5.3%)	

Tumor size (pT)	T1	129 (29.6%)	22 (25.9%)	0.6070	15 (23.4%)	0.3858	6 (31.6%)	0.6489
						
	T2	268 (61.6%)	58 (68.2%)		45 (70.3%)		13 (68.4%)	
						
	T3	34 (7.8%)	4 (4.7%)		3 (4.7%)		0	
						
	Missing	4 (0.9%)	1 (1.2%)		1 (1.6%)		0	

Number of positive lymph nodes	1-3	230 (52.9%)	42 (49.4%)	0.5587	34 (53.1%)	1.0000	8 (42.1%)	0.4825
						
	> = 4	205 (47.1%)	43 (50.6%)		30 (46.9%)		11 (57.9%)	

Histopathological grade	G1	34 (7.8%)	2 (2.3%)	< 0.0001	1 (1.6%)	2.977E-04	1 (5.3%)	0.0078
						
	G2	208 (47.8%)	22 (25.9%)		18 (28.1%)		3 (15.8%)	
						
	G3	186 (42.7%)	60 (70.6%)		44 (68.8%)		15 (79.0%)	
						
	Missing	7 (1.6%)	1 (1.2%)		1 (1.6%)		0	

ER/PgR status	ER+/PgR+	322 (74.0%)	49 (57.6%)	< 0.0001	42 (65.6%)	0.0415	6 (31.6%)	1.424E-04
						
	ER+/PgR-	28 (6.4%)	4 (4.7%)		2 (3.1%)		2 (10.5%)	
						
	ER-/PgR-	67 (15.4%)	31 (36.5%)		19 (29.7%)		11 (57.9%)	
						
	Other	18 (4.1%)	1 (1.2%)		1 (1.6%)		0	

HER2 status	HER2 -	350 (80.4%)	64 (75.3%)	0.1848	48 (75.0%)	0.2145	15 (79.0%)	0.5460
						
	HER2+	71 (16.3%)	20 (23.5%)		15 (23.4%)		4 (21.0%)	
						
	Missing	14 (3.2%)	1 (1.2%)		1 (1.6%)		0	

IHC subtypes	Luminal A	76 (36.8%)	8 (9.4%)	< 0.0001	6 (9.4%)	0.0155	2 (10.5%)	1.592E-04
						
	Luminal B	270 (62.1%)	45 (52.9%)		38 (59.4%)		6 (31.6%)	
						
	HER2	25 (5.7%)	7 (8.2%)		4 (6.3%)		2 (10.5%)	
						
	Triple-negative	42 (9.6%)	24 (28.2%)		15 (23.4%)		9 (47.4%)	
						
	Missing	22 (5.0%)	1 (1.2%)		1 (1.6%)		0	

*Any non-synonymous variation in exon 5 to 8. **P value-chi-square test: comparison of wild-type p53 and p53 mutated. ***P value-Fisher's exact test: comparison of wild-type p53 and p53 mutation (missense or truncating). ****Of 85 p-53 mutations, 83 were classified as mutated (N = 64) or truncating (N = 19), and 2 were not subclassified (see Methods section). ER, estrogen receptor; IHC, immunohistochemistry; PgR, progesterone receptor.

### Prognostic value of p53 mutations

**Figure 1:**
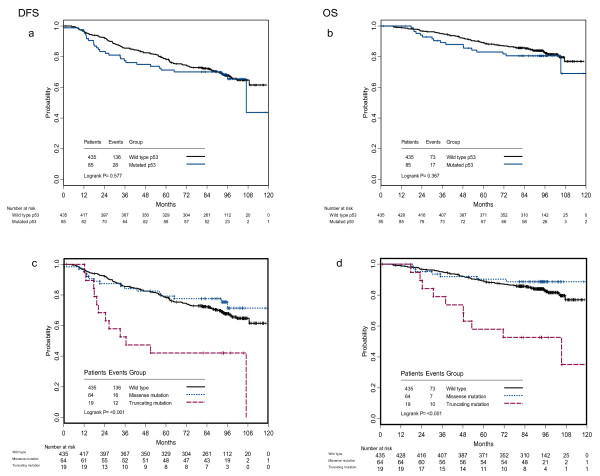
**Prognostic value of p53 mutations**. Survival in p53 wild-type versus p53 mutated cases, **(a) **DFS and **(b) **OS. Survival in p53 wild-type versus missense versus truncated mutations, **(c) **DFS and **(d) **OS. DFS, disease-free survival; OS, overall survival.

At an 8-year median follow-up, the number of DFS events in the entire BIG 02-98 population was 916 of 2887 patients (32%). In this p53 substudy cohort, the number of DFS events was 164 of 520 patients (32%). Survival curves (DFS and OS) for patients included in this p53 substudy are shown in Figure 1. There was no statistically significant difference in DFS or OS based on p53 mutated status. However, when subclassifying mutations, truncating mutations but not missense mutations were found to be associated with a significant reduction in DFS and OS (*P *< 0.001). Further stratification of missense mutations, based on their transactivation capacities or location in the 3D structure of p53 protein, did not reveal classes of mutations with different prognostic value (data not shown). Thus, in univariate analysis only p53 truncating mutations were associated with poor survival.

Multivariate DFS analysis was performed including patient characteristics (age, menopausal status, BMI), tumor characteristics (tumor size, number of positive lymph nodes, histopathological type, histological grade, IHC-defined subtypes, p53 mutation status) and treatment characteristics (mastectomy, radiotherapy, use of tamoxifen, chemotherapy arm (A+AC versus A-T+AT arms), chemotherapy schedule (sequential or concurrent)). The presence of p53 truncating mutations was associated with an increased risk of recurrence (HR = 3.21, 95% CI 1.74 to 5.94, *P *= 0.0002) compared to the absence of mutation within exons 5 to 8. In this multivariate model, only p53 truncating mutations, number of positive lymph nodes (≥ 4 nodes: HR = 1.99, 95% CI: 1.44 to 2.76, *P *< 0.0001) and the HER2 subtype (HR = 2.36, 95% CI 1.21 to 4.60, *P *= 0.0122) had independent prognostic value. (See Table 4.)

**Table 4:** Multivariate Cox proportional hazards models of disease-free survival.

	HR (95% CI)	*P *value
p53 protein status		

Wild-type p53	1	

Missense	0.773 (0.457-1.308)	0.3366

Truncating	3.213 (1.740-5.935)	0.0002

Number of positive lymph nodes		

1-3	1	

≥ 4	1.992 (1.439-2.758)	< 0.0001

IHC subtypes		

Luminal A	1	

Luminal B	1.237 (0.768-1.992)	0.3812

HER2-positive	2.355 (1.205-4.603)	0.0122

Triple-negative	1.086 (0.580-2.032)	0.7965

CI, confidence index; IHC, immunohistochemistry; HR, hazard ratio.

Similar results were obtained for OS. In a multivariate OS analysis, p53 truncating mutations were associated with poor OS compared to the absence of mutation within exons 5 to 8 (HR = 2.75, 95% CI: 1.50 to 5.04, *P *= 0.0011).

### Predictive value of p53 status

**Figure 2:**
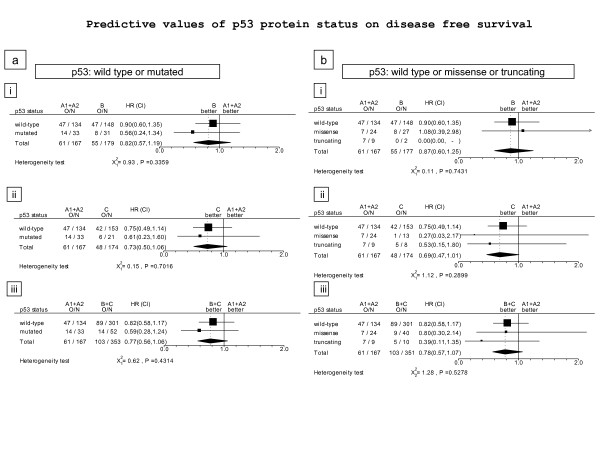
**Value of p53 mutations in predicting DFS benefit from adding docetaxel to control anthracycline-based therapy**. **(a) **Wild-type p53 versus mutant p53 protein. **(b) **p53 wild-type versus missense mutant versus truncated mutant. Treatment comparisons were made between **(i) **anthracycline control arms (A+AC) and sequential docetaxel (A-T); **(ii) **anthracycline control arms (A+AC) and concurrent docetaxel (AT); and **(iii) **anthracycline control arms (A+AC) and combined docetaxel arms (A-T+AT). A, doxorubicin; C, cyclophosphamide; DFS, disease-free survival; T, docetaxel.

In keeping with efficacy results in the entire BIG 02-98 population, this substudy population showed a favorable trend but not a significant benefit in DFS from the addition of docetaxel (HR = 0.77, 95% CI: 0.56 to 1.06). Analyses of the predictive value of p53 status for prediction of improvement in DFS from addition of docetaxel are shown in Figure 2a (p53 wild-type and mutant) and Figure 2b (p53 wild-type, missense and truncating mutations). None of these predictive analyses were statistically significant.

## Discussion

In this retrospective exploratory study performed on samples collected in the context of the prospective BIG 02-98 randomized phase III clinical trial, we show that p53 truncating mutations have a significant independent prognostic value in node-positive breast cancer patients treated with adjuvant chemotherapy. However, no significant value for p53 status in predicting response to docetaxel therapy was found in this cohort.

*TP53 *mutations were detected in a minority of patients (16.3%). Most mutations (75%) were missense, while the remaining (22%) were truncating. The prevalence and type of *TP53 *mutations found are similar with previous studies that have restricted their analysis to exons 5 to 8 and used DNA as starting material (73% of missense mutation in IARC *TP53 *Database, R15). The presence of mutated p53 was associated with patient characteristics of increased age and postmenopausal status, and tumor characteristics of ductal morphology, higher grades and ER/PR negativity, in agreement with previous studies [5,34,35]. The highest rates of mutations were seen in the HER2 and triple-negative subtypes, as reported in other studies [12,36,37].

Most mutations were found at classical hotspot codons and were loss of function mutations as assessed in yeast functional assays [17]. The only exception was a specific hotspot at codon 259, which was found in six samples. The resulting mutation, p.D259N, has been infrequently reported in human cancers (only 14 occurrences in the IARC *TP53 *Database, none in breast cancers [19]) and retains partial activity in yeast functional assays [17]. We ruled out PCR contamination and technical causation. The origin of this mutation remains to be determined.

Truncating mutations had an independent prognostic value against a large range of clinical and molecular variables. These results confirm and extend those obtained in previous studies, including our own performed on a large consecutive series of breast cancers [5]. Interestingly, missense mutations were not associated with poor survival in the current study. Discrimination between missense mutations according to location inside or outside DNA-binding motifs, mutations that retain or do not retain transactivation activities, or have dominant-negative effects did not differentiate missense mutations with poorer survival (data not shown). This is in contrast with our previous study, which showed that missense mutations within DNA-binding motifs (but not those outside these motifs) and non-missense mutations were associated with poor prognosis [5]. There was no obvious difference between the type of missense mutation found in this series and earlier studies to explain these discrepant results. Inconsistency of results regarding the prognostic value of missense mutations across studies may be due to the fact that missense mutations may retain some activity, beyond the specific activity detectable in a particular study. This is in contrast to more consistent results for truncating mutations, which are true loss of function mutations.

The multivariate model for the prognostic value of p53 included many variables, notably it included BMI- and IHC-defined molecular subtypes. BMI was included as obesity was found to have an independent poor prognostic value in this trial as well as in other studies [38,39]. In this substudy however, BMI had no independent prognostic value. The independent predictors of poor DFS and OS were p53 truncating mutations, high number of positive lymph nodes and the HER2 subtype. It is of note that BIG 02-98 was conducted prior to the use of adjuvant HER2-targeted therapies. The prognostic value of HER2 subtype is thus independent of HER2-targeted treatment in these patients. Interestingly, the triple-negative subtype did not have prognostic value in the univariate analysis and had no independent prognostic value. Many studies in early breast cancer have reported that the triple-negative subtype is particularly aggressive and associated with a high rate of recurrence and early death. A possible explanation for the lack of adverse outcomes in the triple-negative subtype in the current study may be the limited sample size.

In this study, p53 mutations had no significant predictive value. This study adds to the conflicting body of clinical evidence regarding the potential clinical role of p53 as a single biomarker for prediction of chemotherapy response. Other studies assessing *TP53 *gene sequence have presented data in support of reduced anthracycline activity in *TP53 *mutated tumors [9-11]. However, the predictive correlations were not robust enough for *TP53 *gene status, as a single biomarker, to be considered a clinical tool to guide treatment decisions. Three recent neoadjuvant studies in early breast cancer have reported a predictive role for *TP53 *mutations. In a phase II Xeloda in Neoadjuvant (XeNa) trial assessing capecitabine and docetaxel, with trastuzumab if HER2-positive, a secondary study endpoint was evaluation of *TP53 *status and response to chemotherapy [12]. *TP53 *mutations, detected by AmpliChip p53 (Roche Diagnostics, Pleasanton, CA, USA), correlated significantly with improved pathological complete response rate compared with *TP53 *wild-type (30% versus 10%, respectively, *P *= 0.0032). In a study using dose-dense epirubicin-cyclophosphamide, *TP53 *mutations were detected using a yeast functional assay. Pathological complete responses, which occurred in 19% (15 of 80 patients), were restricted to tumors with a *TP53 *mutation (28 of 80 patients) [40]. In another small study that included only triple-negative breast cancers patients treated with neoadjuvant cisplatin, nonsense and frameshift *TP53 *mutations were associated with good response to treatment [41]. The lack of a control arm in these three studies limits interpretation of prognosis versus prediction, and of general versus specific agent chemosensitivity.

An important recent study is the neoadjuvant phase III EORTC 10994/BIG 00-01 trial which is the only study to be prospectively powered to assess the value of p53 status for prediction of docetaxel benefit [14]. Using an RNA-based technique that detects functionally important p53 mutations, the prognostic role of p53 was confirmed, but p53 was not predictive of response or resistance to docetaxel.

The negative results from the prospective EORTC study and our BIG 02-98 substudy, with a background of inconsistent results, suggest that p53 status as an isolated marker may be inadequate to predict treatment response to docetaxel. p53 is not a drug target, but rather a surrogate measure for cellular capacity for apoptosis, and cell proliferation control. The complexity of p53 function, post-transcriptional effects and up- and down-stream signaling pathway cross-talks may limit the power of p53 status as a sole biomarker, in particular for treatments that combine drugs that have different mode of action towards p53.

A strength of the current study is the biological distinction of nonsynonymous mutations as missense (in and out of DNA-binding motifs) and truncating. Grouping all mutations together for assessment of outcomes assumes homogeneity in the impact of different mutations. Use of a dichotomous classification did not identify a prognostic or predictive correlation, whereas the more specific classification system revealed the independent prognostic significance value of the truncating mutations. A limitation of this approach however, is that such a cohort contain few patients, limiting to some degree the confidence with which conclusions may be drawn for the subsets.

The presumption of a homogeneous role of p53 across diverse biological subgroups may also account for inconsistent findings. In distinct breast cancer subgroups, p53 mutations may have variable prevalence, and impact differently on prognosis and treatment sensitivity. As part of the I-SPY neoadjuvant initiative (CALGB 150007), a recent study reported *TP53 *status determined by gene chip technology and sequencing in women treated with anthracycline- then taxane-based therapy [42]. In the luminal A subtype, 14 of 48 patients has a *TP53 *mutation, and there was no differential efficacy of either treatment based on *TP53 *status. In contrast, *TP53 *mutations had a much higher prevalence in the luminal B, basal-like and HER2 subgroups, in whom, anthracycline efficacy was independent of *TP53 *status and taxane efficacy was greater in the presence of wild-type *TP53*. In the current study, mutated p53 was indeed more prevalent in HER2 and triple-negative subtypes. However, small numbers precluded analyses by subgroups.

A critical issue in the clinical translation of p53 is the impact of gene variations on p53 protein function. Herein the classification of p53 mutations was based on estimated downstream impact on protein sequence. A reported alternative for identification of 'functional' loss of wild-type p53 is a p53 transcriptional fingerprint. Several gene expression signatures have been reported that distinguish between wild-type and mutant p53 [36,43,44]. Interestingly some *TP53 *wild-type tumors expressing the mutation-associated 32-gene signature behaved aggressively, while some *TP53 *mutant tumors lacking the signature had favorable outcome [43]. In another recent report, a 39-gene and a 30-gene p53 signature were developed in ER-positive and -negative breast cancer, respectively [45]. It is of note that there was no overlap in genes across the two signatures. The ER-positive p53 signature in ER-positive disease was predictive of poor prognosis and increased chemosensitivity. The ER-negative p53 signature in ER-negative disease was associated with improved prognosis, but had no prediction of treatment response. Downstream functional assessment of p53 status may also capture the impact of other p53 variables that may influence tumor outcome and treatment sensitivity, such as p53 codon polymorphisms [46] and p53 protein isoforms [47]. Further studies using downstream functional assessment of p53 status are thus needed to better understand the impact of p53 mutations on tumor phenotype.

The current study has limitations. This is a retrospective exploratory analysis, which was not considered in the initial statistical trial design or trial sample size determination. In the trial, all patients received polychemotherapy so it is impossible to determine interactions between single-agent activity and p53 status. Only exons 5 to 8 of *TP53 *gene were screened for mutation due to the limited amount of DNA available. According to previous studies, up to 20% of mutations may fall outside these exons (IARC *TP53 *Database, R15). To evaluate the number of misclassified samples due to this partial sequencing, 84 samples were successfully sequenced for exon 4, the next most mutated exon in breast cancers (IARC *TP53 *Database, R15). One truncating and 13 missense mutations were found. Since all missense mutations retained transactivation activity, only 1 of 84 (1.2%) was considered a true deleterious mutation. As mutations in other exons are expected to be rare (less than 1% of mutations reported in breast cancers fall outside exons 4 to 8), we estimate that less than 2% of samples may have been misclassified as wild-type in our series.

## Conclusions

Despite an abundant literature on the prognostic and predictive value of p53 status in breast cancer, a limited number of studies have used gene sequencing to assess p53 status and even fewer studies have been performed in the context of controlled clinical trials. The results confirm that loss of wild-type p53 through protein truncating mutations is associated with poor prognosis. Our results point to differences in the prognostic value of different types of mutations and to the difficulty of assessing the predictive value of a molecular marker in treatment regimens that combine drugs with different modes of action regarding this marker.

## Abbreviations

A: doxorubicin; BIG: Breast International Group; BMI: body mass index; C: cyclophosphamide; CI: confidence interval; DFS: disease-free survival; EIO: European Institute of Oncology; ER: estrogen receptor; F: 5-fluorouracil; FFPE: formalin-fixed paraffin-embedded; FISH: fluorescence in situ hybridization; HER2: human epidermal growth factor receptor 2; HR: hazard ratio; IARC: International Agency for Research on Cancer; IHC: immunohistochemistry; IJB: Institut Jules Bordet; M: methotrexate; OS: overall survival; PCR: polymerase chain reaction; PgR: progesterone receptor; T: docetaxel.

## Competing interests

Prudence A. Francis: conference travel support from Sanofi-Aventis. Martine Piccart-Gebhart: Institut Jules Bordet has numerous contracts in which Dr. Piccart-Gebhart is an investigator (Sanofi-Aventis, Amgen, Bayer, Bristol-Myers Squibb, Roche, Glaxo SK, Boehringer, PharmaMar). All other authors declare they have no competing interests.

## Authors' contributions

LF-C participated in the study design; undertook *TP53 *gene mutation screening; and participated in the drafting of the manuscript. CO participated in the study design; performed data analysis; and drafted the manuscript. PF-L participated in *TP53 *gene mutation screening. KS participated in *TP53 *gene mutation screening. EQ participated in the study design; performed the statistical analysis; and drafted the manuscript. MB participated in the design of the study and performed the statistical analysis. MSD participated in the study design and coordination; performed data analysis; participated in the statistical analysis; and drafted the manuscript. EDA participated in the study design and coordination; performed data analysis; and drafted the manuscript. PH has given final approval of the version to be published. PD performed the central pathology analyses, DNA extraction, slide review, IHC and fluorescence in situ hybridization (FISH). DL supervised the central laboratory; and tissue and slide preparation. PAF conceived of the original trial design and co-ordination; performed data analysis; and drafted the manuscript. JC conceived of the original trial design and co-ordination; performed data analysis; and drafted the manuscript. MP-G conceived of the original trial design and co-ordination; performed data analysis; and drafted the manuscript. GV participated in the study design and coordination; undertook central pathology analyses, slide review, IHC and fluorescence in situ hybridization (FISH); performed DNA extraction, performed data analysis; and drafted the manuscript. ADL conceived the original trial design and co-ordination; performed data analysis; and drafted the manuscript. MO designed and coordinated the p53 sub-study, supervised *TP53 *sequencing, performed analysis and interpretation of data; and drafted the manuscript. All authors read and approved the final manuscript.

## Supplementary Material

Additional file 1 **Table S1, Characteristics of patients analyzed for *TP53 *mutations compared with the entire BIG-02-98 cohort**. From BIG 02-98, 666 patients with centrally submitted FFPE primary tissue and sufficient remaining tumor tissue for *TP53 *gene analysis were selected for the p53 biomarker study. Of these, 520 tumors were successfully analyzed for exons 5-8. This table contains baseline patient and tumor characteristics, showing that the substudy population was representative of the entire BIG-02-98 population. Click here for file

Additional file 2 **Table S2, Immunohistochemistry (IHC) subtypes as defined in this study**. Four breast cancer subtypes (luminal A, luminal B, HER2-positive and triple-negative) were defined using central laboratory defined parameters (ER, PgR, HER2, grade and Ki-67). Click here for file

Additional file 3 **Figure S1, Representativeness of the p53 cohort: disease-free survival for patients included in the p53 substudy and patients not included in the p53 substudy from the BIG 02-98 trial**. Kaplan-Meier curve confirming similar disease-free survival for BIG 02-98 patients included and not included in this substudy. Click here for file

Additional file 4 **Table S3, *TP53 *gene variations found in the p53 substudy**. From 520 analyzed tumors, 96 variations within exon 5 to 8 were found in 90 samples (90 of 520, 17%). This table lists *TP53 *gene variation data for the 90 samples. Eighty-five patients had only one variation, four patients (ID: 10120, 10202, 42618, 62514) had two variations, and one patient had three variations (ID: 22205). Click here for file
