# Supplementary material for: Prognostic and predictive value of TP53 mutations in node-positive breast cancer patients treated with anthracycline- or anthracycline/taxane-based adjuvant therapy: results from the BIG 02-98 phase III trial
Source: Breast Cancer Res. 2012 May 2;14(3):R70. doi: 10.1186/bcr3179 (PMC3446332; doi:10.1186/bcr3179)
Supplement: Additional file 1 — Table S1, Characteristics of patients analyzed for TP53 mutations compared with the entire BIG-02-98 cohort. From BIG 02-98, 666 patients with centrally submitted FFPE primary tissue and sufficient remaining tumor tissue for TP53 gene analysis were selected for the p53 biomarker study. Of these, 520 tumors were successfully analyzed for exons 5-8. This table contains baseline patient and tumor characteristics, showing that the substudy population was representative of the entire BIG-02-98 population. [file bcr3179-S1.PDF]

Additional file 1, Table S1.

Characteristics of patients analyzed for *TP53* mutations compared with the entire BIG-02-98 cohort.

		p53 study N=520	BIG-02-98 N=2887
Age	Median	49	49
	Range	26-70	21-70
	<35	34 (6.5%)	195 (6.8%)
	35-49	255 (49.0%)	1346 (46.6%)
	50-65	213 (41.0%)	1231 (42.6%)
	>65	18 (3.5%)	115 (4.0%)
Menopausal status	Pre-menopausal	286 (55.0%)	1552 (53.8%)
	Post-menopausal	206 (39.6%)	1173 (40.6%)
	missing	28 (5.4%)	162 (5.6%)
Histopathology	Infiltrating ductal ca.	424 (81.5%)	1045 (79.5%)
	Infiltrating lobular ca.	53 (10.2%)	174 (13.2%)
	Other	43 (8.3%)	96 (7.3%)
Histopathologic grade	G1	50 (9.6%)	91 (6.9%)
	G2	242 (46.5%)	566 (43.0%)
	G3	215 (41.3%)	609 (46.3%)
	missing	13 (2.5%)	49 (3.7%)
Number of positive lymph nodes	1-3	272 (52.3%)	1567 (54.3%)
	> 4	248 (47.7%)	1320 (45.7%)
ER/PR status	ER+/PR+	296 (56.9%)	1482 (51.3%)
	ER+/PR-	48 (9.2%)	342 (11.8%)
	ER-/PR-	114 (21.9%)	685 (23.7%)
	other	62 (11.9%)	378 (13.1%)
IHC subtypes	Luminal A	84 (16.1%)	294 (10.2%)
	Luminal B	315 (60.6%)	1034 (35.8%)
	HER2	32 (6.2%)	149 (5.2%)
	Triple negative	66 (12.7%)	300 (10.2%)
	missing	23 (4.4%)	1110 (38.4%)
Hormonotherapy (HT) usage	Received HT	394 (75.8%)	2126 (73.6%)
