# Supplementary material for: Prognostic and predictive value of TP53 mutations in node-positive breast cancer patients treated with anthracycline- or anthracycline/taxane-based adjuvant therapy: results from the BIG 02-98 phase III trial
Source: Breast Cancer Res. 2012 May 2;14(3):R70. doi: 10.1186/bcr3179 (PMC3446332; doi:10.1186/bcr3179)
Supplement: Additional file 2 — Table S2, Immunohistochemistry (IHC) subtypes as defined in this study. Four breast cancer subtypes (luminal A, luminal B, HER2-positive and triple-negative) were defined using central laboratory defined parameters (ER, PgR, HER2, grade and Ki-67). [file bcr3179-S2.PDF]

Additional file 2, Table S2.

IHC subtypes as defined in this study.

Subtype (IHC class)	Code	Rules
Luminal A	1	ER positive (≥ 1% of cells staining positive)[a] **and**<br>PgR positive (≥ 1% of cells staining positive), **and**<br>Ki67 <14%[b,c] (assessed on whole section), **and**<br>HER2 negative cases (0, 1+, 2+, FISH negative)
Luminal B	2	ER positive and PgR negative independently of Grade, HER2 and Ki-67,<br>**or**<br>ER positive[a] and PgR positive, **with**<br>Ki-67 ≥14%, **and/or**<br>HER2 positive (3+ or 2+ FISH +), **and/or**<br>Grade 3
HER-2 positive	3	ER negative **and** PgR negative **and** HER2 positive
Triple Negative	4	ER negative **and** PgR negative **and** HER2 negative

[a] ER negative and PgR positive cases were considered to be a false negative ER result, and thus were categorized as ER and PgR positive

[b] based on Cheang et al, 2009 [30]

[c] if Ki-67 was missing, grade 1 was considered a surrogate for low Ki-67; if Ki-67 was missing and grade 2, the case was considered not evaluable
