# Supplementary material for: Prognostic and predictive value of TP53 mutations in node-positive breast cancer patients treated with anthracycline- or anthracycline/taxane-based adjuvant therapy: results from the BIG 02-98 phase III trial
Source: Breast Cancer Res. 2012 May 2;14(3):R70. doi: 10.1186/bcr3179 (PMC3446332; doi:10.1186/bcr3179)
Supplement: Additional file 3 — Figure S1, Representativeness of the p53 cohort: disease-free survival for patients included in the p53 substudy and patients not included in the p53 substudy from the BIG 02-98 trial. Kaplan-Meier curve confirming similar disease-free survival for BIG 02-98 patients included and not included in this substudy. [file bcr3179-S3.PDF]

Additional file 3, Figure S1

Representativeness of the p53 cohort: disease-free survival for patients included in the p53 substudy and patients not included in the p53 substudy from the BIG 02-98 trial

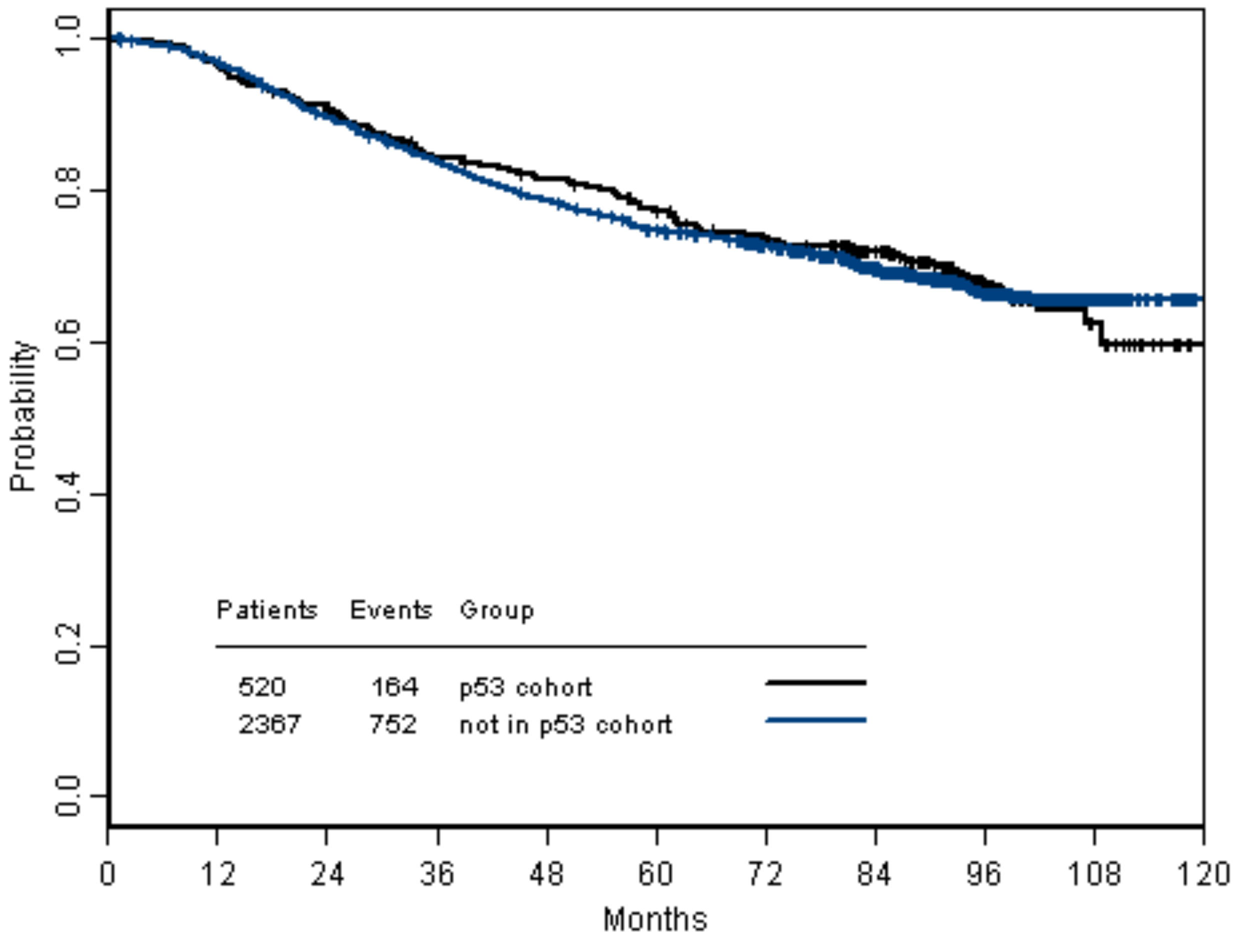

Number at risk

	0	12	24	36	48	60	72	84	96	108	120
p53	520	499	467	431	412	387	361	313	135	22	1
not in p53	2367	2278	2098	1950	1826	1717	1623	1267	510	79	3
