# Supplementary material for: Prognostic and predictive value of TP53 mutations in node-positive breast cancer patients treated with anthracycline- or anthracycline/taxane-based adjuvant therapy: results from the BIG 02-98 phase III trial
Source: Breast Cancer Res. 2012 May 2;14(3):R70. doi: 10.1186/bcr3179 (PMC3446332; doi:10.1186/bcr3179)
Supplement: Additional file 4 — Table S3, TP53 gene variations found in the p53 substudy. From 520 analyzed tumors, 96 variations within exon 5 to 8 were found in 90 samples (90 of 520, 17%). This table lists TP53 gene variation data for the 90 samples. Eighty-five patients had only one variation, four patients (ID: 10120, 10202, 42618, 62514) had two variations, and one patient had three variations (ID: 22205). [file bcr3179-S4.PDF]

Additional file 4, Table S3.

TP53 gene variations found in the p53 substudy.

Sample_ID	p53 status	c_description	p_description	Effect	TransactivationClass [(a)]	EffectGroup3[(b)]
10111	mutated	c.506T>C	p.M169T	missense	functional	1
10120	mutated	c.762C>T	p.I254I	silent	NA	0
		c.865C>T	p.L289F	missense	supertrans	2
10193	mutated	c.538G>A	p.E180K	missense	partially functional	1
10202	mutated	c.824G>T	p.C275F	missense	non-functional	1
		c.505A>G	p.M169V	missense	partially functional	1
10301	mutated	c.455_456ins1	p.?	FS	NA	4
10401	mutated	c.395A>G	p.K132R	missense	non-functional	1
10701	mutated	c.775G>A	p.D259N	missense	partially functional	2
10719	mutated	c.395A>G	p.K132R	missense	non-functional	1
10726	mutated	c.818G>A	p.R273H	missense	non-functional	1
10738	mutated	c.836G>A	p.G279E	missense	non-functional	1
10743	mutated	c.584T>C	p.I195T	missense	non-functional	2
10817	mutated	c.853G>A	p.E285K	missense	non-functional	1
11312	mutated	c.702C>A	p.Y234X	nonsense	NA	4
11609	mutated	c.797G>A	p.G266E	missense	non-functional	2
12803	mutated	c.600_601ins1	p.?	FS	NA	4
12809	mutated	c.722C>T	p.S241F	missense	non-functional	1
12810	mutated	c.513G>T	p.E171D	missense	partially functional	1
13104	mutated	c.775G>A	p.D259N	missense	partially functional	2
14205	mutated	c.497C>G	p.S166X	nonsense	NA	4
14212	mutated	c.434T>C	p.L145P	missense	non-functional	2
14418	mutated	c.775G>A	p.D259N	missense	partially functional	2
14425	mutated	c.731G>A	p.G244D	missense	non-functional	1
14431	mutated	c.646G>A	p.V216M	missense	non-functional	2
15125	mutated	c.733G>T	p.G245C	missense	non-functional	1
15207	mutated	c.743G>A	p.R248Q	missense	non-functional	1

15601	mutated	c.697del1	p.?	FS	NA	4
15804	mutated	c.878G>A	p.G293E	missense	functional	2
15815	mutated	c.844C>G	p.R282G	missense	non-functional	1
15905	mutated	c.625_626del2	p.?	FS	NA	4
16007	mutated	c.596G>T	p.G199V	missense	non-functional	2
16102	mutated	c.514G>A	p.V172I	missense	functional	1
16513	mutated	c.422G>A	p.C141Y	missense	non-functional	2
17014	Wild-type	c.534C>T	p.H178H	silent	NA	0
17406	mutated	c.637C>T	p.R213X	nonsense	NA	4
17711	mutated	c.413C>T	p.A138V	missense	partially functional	2
17801	mutated	c.637C>T	p.R213X	nonsense	NA	4
22205	Wild-type	c.782+26C>T	p.?	intronic	NA	0
		c.782+10C>T	p.?	intronic	NA	0
		c.673-17C>T	p.?	intronic	NA	0
22401	mutated	c.469G>T	p.V157F	missense	non-functional	2
22408	mutated	c.839G>C	p.R280T	missense	non-functional	1
22419	mutated	c.850A>C	p.T284P	missense	non-functional	1
22423	mutated	c.586C>G	p.R196G	missense	partially functional	2
26205	mutated	c.637C>T	p.R213X	nonsense	NA	4
30208	mutated	c.742C>G	p.R248G	missense	non-functional	1
30225	mutated	c.781A>T	p.S261C	missense	functional	2
34122	mutated	c.659A>G	p.Y220C	missense	non-functional	2
34125	mutated	c.743G>A	p.R248Q	missense	non-functional	1
34141	mutated	c.488A>G	p.Y163C	missense	non-functional	2
38315	mutated	c.843_844ins1	p.?	FS	NA	4
38325	mutated	c.574C>T	p.Q192X	nonsense	NA	4
38422	mutated	c.730G>T	p.G244C	missense	non-functional	1
42214	mutated	c.413C>T	p.A138V	missense	partially functional	2
42221	mutated	c.775G>A	p.D259N	missense	partially functional	2
42605	mutated	c.517G>T	p.V173L	missense	non-functional	1
42609	mutated	c.747G>T	p.R249S	missense	non-functional	1

42618	mutated	c.916C>T	p.R306X	nonsense	NA	4
		c.486C>T	p.I162I	silent	NA	0
43023	mutated	c.637C>T	p.R213X	nonsense	NA	4
46103	Wild-type	c.782+9C>T	p.?	intronic	NA	0
46205	mutated	c.530C>T	p.P177L	missense	non-functional	1
46207	mutated	c.524G>A	p.R175H	missense	non-functional	1
50310	mutated	c.524G>A	p.R175H	missense	non-functional	1
50317	mutated	c.742C>T	p.R248W	missense	non-functional	1
50371	mutated	c.428T>G	p.V143G	missense	non-functional	2
50380	Wild-type	c.390C>T	p.L130L	silent	NA	0
54704	mutated	c.637C>T	p.R213X	nonsense	NA	4
55207	mutated	c.577C>T	p.H193Y	missense	non-functional	1
55301	mutated	c.526T>C	p.C176R	missense	non-functional	1
58301	mutated	c.757_758ins1	p.?	FS	NA	4
58303	Wild-type	c.673-12G>T	p.?	intronic	NA	0
58307	mutated	c.856G>A	p.E286K	missense	non-functional	1
62506	mutated	c.775G>A	p.D259N	missense	partially functional	2
62514	mutated	c.782+1G>A	p.?	splice	NA	4
		c.783-12T>C	p.?	intronic	NA	0
63410	mutated	c.422G>A	p.C141Y	missense	non-functional	2
63811	mutated	c.747G>T	p.R249S	missense	non-functional	1
67027	mutated	c.832C>T	p.P278S	missense	non-functional	1
70101	mutated	c.743G>C	p.R248P	missense	non-functional	1
70111	mutated	c.659A>G	p.Y220C	missense	non-functional	2
70201	mutated	c.733del1	p.?	FS	NA	4
70214	mutated	c.574C>T	p.Q192X	nonsense	NA	4
70215	mutated	c.817C>G	p.R273G	missense	non-functional	1
70219	mutated	c.460G>A	p.G154S	missense	partially functional	2
78102	mutated	c.524G>A	p.R175H	missense	non-functional	1
78141	mutated	c.535C>T	p.H179Y	missense	partially functional	1
78149	mutated	c.461G>T	p.G154V	missense	non-functional	2

78150	mutated	c.396G>C	p.K132N	missense	non-functional	1
78172	mutated	c.488A>G	p.Y163C	missense	non-functional	2
78187	mutated	c.734G>A	p.G245D	missense	non-functional	1
82101	mutated	c.818G>A	p.R273H	missense	non-functional	1
82214	mutated	c.537T>G	p.H179Q	missense	non-functional	1
82303	mutated	c.438G>A	p.W146X	nonsense	NA	4
90404	mutated	c.775G>A	p.D259N	missense	partially functional	2

NA: not applicable

(a) and (b) Annotations available in the IARC TP53 Database

(a) Transactivation activity of missense mutations based on functional assays in yeast; (b) Missense mutations classification: 1, missense mutations located in DNA-binding motifs; 2, missense mutations located outside DNA-binding motifs; 4, truncating mutations;
